# Protein Sulfhydryl Group Oxidation and Mixed-Disulfide Modifications in Stable and Unstable Human Carotid Plaques

**DOI:** 10.1155/2013/403973

**Published:** 2013-02-04

**Authors:** Antonio Junior Lepedda, Angelo Zinellu, Gabriele Nieddu, Elisabetta Zinellu, Ciriaco Carru, Rita Spirito, Anna Guarino, Pierina De Muro, Marilena Formato

**Affiliations:** ^1^Dipartimento di Scienze Biomediche, Unviversità di Sassari, Via Muroni 25, 07100 Sassari, Italy; ^2^Centro Cardiologico “F. Monzino”, IRCCS, 20138 Milano, Italy

## Abstract

*Objectives*. Oxidative stress has been implicated in the outcome of atherosclerotic plaques. However, at present, no data are available neither on the degree of plaque protein sulfhydryl groups oxidation nor on its relationship with plaque vulnerability. We investigated the entity of protein-SH oxidative modifications, focusing on low molecular weight thiols adduction, in human carotid plaque extracts in relation to plaque stability/instability. *Methods*. Plaque stability/instability was histologically assessed. The extent of protein-SH oxidative modifications was established by a differential proteomic approach on fluorescein-5-maleimide-labeled plaque extracts and corresponding plasma samples from 48 endarterectomized patients. The analysis on protein thiolation was performed by capillary zone electrophoresis. *Results*. We observed a higher protein-SH oxidation of both plasma-derived and topically expressed proteins in unstable plaques, partly due to higher levels of S-thiolation. Conversely, in plasma, none of the investigated parameters discriminated among patients with stable and unstable plaques. *Conclusions*. Our results suggest the presence of a more pronounced oxidative environment in unstable plaques. Identifying specific oxidative modifications and understanding their effects on protein function could provide further insight into the relevance of oxidative stress in atherosclerosis.

## 1. Introduction

Plaque rupture and thrombosis are the most important clinical complications in the pathogenesis of acute coronary syndromes and peripheral vascular disease [1]. Although the exact mechanisms underlying plaque vulnerability are not completely clear, it is generally held that plaque instability is characterized by a pronounced proteolytic and proinflammatory environment [2]. In a previous study, we provided evidence for a wide fragmentation of some apolipoproteins and arterial proteoglycans and for a pro-inflammatory microenvironment in unstable and much less in stable endarterectomy carotid plaques [3]. Recently, by applying proteomics to the study of carotid plaque vulnerability, we identified a panel of proteins, differently expressed in stable/unstable lesions, with prooxidant and proinflammatory potentials according to our current understanding of the molecular basis of the atherosclerotic process [4]. *In situ* oxidative events may have important functional consequences on protein metabolic fate as well as on their bioactivity and antigenic properties. In this respect, oxidised LDL is readily internalized by macrophages through the so-called “scavenger receptor” pathway [5]. These early modifications could initiate and/or contribute to atherogenesis, mainly when an imbalance between oxidant and antioxidant agents takes place. Many enzymes (such as superoxide dismutases, catalase, peroxidases, glutathione-S transferase and reductase, and peroxiredoxins), as well as nonenzymic proteins (transferrin, ferritin, albumin, and aptoglobin), and nonproteinaceous antioxidants (ascorbic acid, uric acid, and *α*-tocopherol) are known to participate in maintaining a reductive environment within the arterial wall [6]. Together with these antioxidants, protein sulfhydryl groups (protein-SH groups, PSH) and low molecular weight thiols (LMW-thiols) are involved in the cell regulation of reactive oxygen species levels. Many proteins and enzymes have cysteine residues in their side chain, and their proton lability makes them chemical hot spots for a wide variety of biochemical interactions such as the reversible reaction of *S*-thiolation (the formation of mixed disulfides among protein thiols and LMW-thiols) [7]. The formation of S-thiolated proteins could be the result of an antioxidant response, and it has been suggested as a possible redox regulation mechanism of protein function [8–10]. So, the reversible covalent modification of some protein cysteine residues may be transitory and have critical modulation effects. It has been suggested that homocysteinylation could activate latent elastolytic metalloproteinase-2 (pro-MMP-2) by disulfide bond formation on the propeptide via the so-called “cysteine switch” mechanism [11]. In this regard, also the S-glutathionylation is thought to play a role [12]. All these evidences have led to the intriguing hypothesis that direct LMW-thiols-mediated matrix metalloproteinases (MMPs) activation could be involved in the extracellular matrix degradation and plaque rupture.

We have previously demonstrated that LDL apolipoprotein B-100 is able to bind all plasma thiols [13, 14] and that human carotid atherosclerotic plaque has different levels and distribution of LMW thiols with respect to plasma [15]. In this respect, the elevated levels of intraplaque glutathione may induce important effects on plaque fate by perturbing the physiological LMW thiol redox state.

The aim of the present work was to investigate the redox status of protein sulfhydryl groups extracted from atherosclerotic plaques in relation to lesion stability. Moreover, we evaluated levels and distributions of both total and protein-bound LMW-thiols to assess the degree of protein mixed-disulfide modification.

## 2. Methods

### 2.1. Patient Population

Proteomic and capillary zone electrophoresis (CZE) analyses were conducted on both carotid plaque specimens and plasma samples from forty-eight patients undergoing carotid endarterectomy, enrolled in a previous study and screened for plaque stability by immunohistochemistry [4]. Informed consent was obtained before enrollment. The study was approved by the local Ethical Committee of the University of Milan in accordance with institution guidelines.

### 2.2. Plaque and Plasma Samples

Tissue extracts from carotid endarterectomy specimens, histologically classified as stable (*n* = 19) or unstable (*n* = 29) plaques, were obtained as previously described [3, 4]. Briefly, the plaque segments were washed in PBS, finely minced, and subjected to protein extraction with a solubilisation buffer (8 M urea, 4% w/v CHAPS, 45 mM Tris), supplemented with 100 *μ*mol/L APMSF, 2 *μ*g/mL KI, and 50 *μ*mol/L leupeptin, under continuous shaking for 1 h at room temperature. Extracts were collected by centrifuging the resulting suspension at 65,000 ×g in a TL-100 Beckman centrifuge for 30 min at 20°C.

Before surgery, blood was collected in Vacutainer tubes containing EDTA. After centrifugation at 1000 ×g at 4°C for 15 minutes, the plasma was separated, supplemented with the aforementioned antiproteolytic agents, and stored at −80°C until analysis.

### 2.3. Proteomic Analysis

Tissue extracts were delipidated and resolubilized as previously described [4].

PSH of both resolubilized plaque extracts and plasma samples were fluorotagged with fluorescein-5-maleimide (F5M) in the dark following the manufacturer instructions (PIERCE Biotechnology). 

8 *μ*g and 50 *μ*g of F5 M-labelled protein were loaded for 1D SDS-PAGE and 2D electrophoresis, respectively. 2D electrophoresis was conducted as already reported [4]. Briefly, IEF was performed using 70 mm, immobilised linear pH 4–8 gradient strips (Nurex srl, Sassari, Italy). IPG strips were rehydrated overnight at 20°C with 50 *μ*g of F5 M-labelled protein diluted in a solubilisation buffer containing 1% w/v DTT and 2% v/v Pharmalyte (pH 3.5–10) and subsequently focused at 50 *μ*A/IPG strip for 22 kVh at 18°C. Once IEF was completed, the strips were equilibrated under continuous shaking for 15 min in 50 mM Tris-HCl buffer containing 6 M urea, 30% v/v glycerol, and 3% w/v SDS with the addition of 1% w/v DTT, followed by an equilibration for 15 min in the same buffer without DTT, but with the addition of 2.5% w/v iodoacetamide. The IPG strips were then sealed with 0.5% low melting point agarose in SDS running buffer at the top of slab gels (8 × 7 × 0.1 cm). SDS-PAGE was performed on 10%T, 3%C polyacrylamide separating gels in a MiniProtean II cell vertical slab gel electrophoresis apparatus (Bio-Rad, Hercules, CA, USA).

Fluorescence images of resolved proteins were acquired by using the Gel Doc XR system (Bio-Rad, Hercules, CA, USA), and, subsequently, gels were stained with Coomassie Brillant Blue G250 (CBB). Images were analysed using Quantity One 4.6.3 software (Bio-Rad, Hercules, CA, USA). Band fluorescence data were normalized for the corresponding intensity after CBB staining. Protein spots identification was performed by peptide mass fingerprinting analysis as reported elsewhere [4].

### 2.4. Capillary Zone Electrophoresis (CZE) Analysis

Total and protein-bound LMW-thiols in plaque extracts and corresponding plasma samples were assayed as previously described [15].

Briefly, for total LMW-thiols assay, 20 *μ*L of extract were treated with 20 *μ*L of internal standard NAC (6 *μ*M) and 4 *μ*L of 10% TBP in DMF (v/v) for 10 min. Then, proteins were precipitated by adding 1 mL of acetonitrile (ACN), and supernatant containing LMW-thiols was dried under vacuum and resolubilized with 100 *μ*L of 30 mM sodium phosphate buffer (pH 12.5) containing 0.08 mM 5-IAF.

For protein-bound thiols assay, 600 *μ*g of delipidated proteins were resolubilised in 200 *μ*L of 1 mM NaOH and 2 *μ*L of NAC (1.5 *μ*mol/L) at 60°C for 30 min. Disulfide bonds were reduced by incubating with 20 *μ*L of 10% TBP in DMF for 10 min, and proteins were precipitated by adding 900 *μ*L of ACN. Then, supernatant was dried under vacuum, and LMW-thiols were derivatized as previously mentioned. 

LMW-thiols measurement was performed by a CE system (P/ACE 5510) equipped with a LIF detector (Beckman, Palo Alto, CA, USA).

Total LMW-thiols were calculated as the sum of free LMW-thiols and protein-bound LMW-thiols.

### 2.5. Statistical Analysis

Differences between stable and unstable plaques were evaluated by either the Student *t-*test, for normally distributed data, or the Mann-Whitney Rank Sum test, for nonparametric ones. Differences with *P* values < 0.05 were considered to be significant.

## 3. Results

### 3.1. PSH Oxidation (Proteomic Analysis)

**Figure 1:**
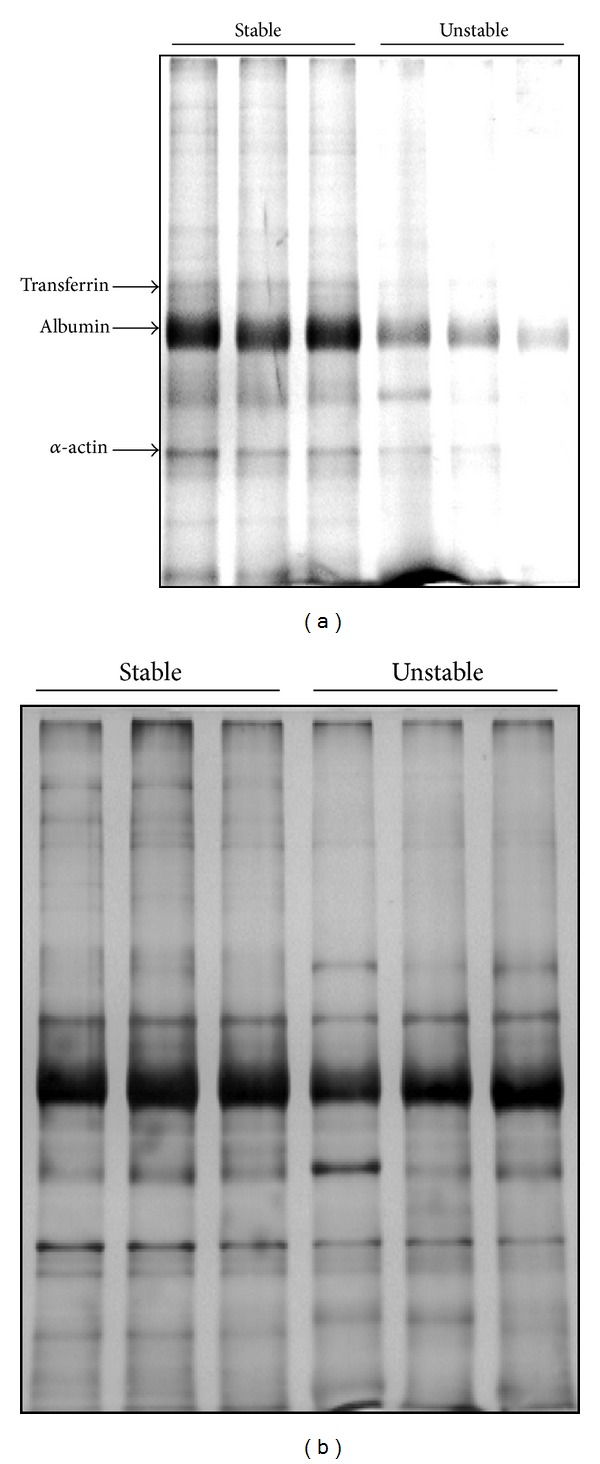
Representative SDS-PAGE patterns of F5M-labeled proteins from stable and unstable plaque extracts (a) and the corresponding CBB staining (b). In (a), inverted fluorescent image is reported.

**Figure 2:**
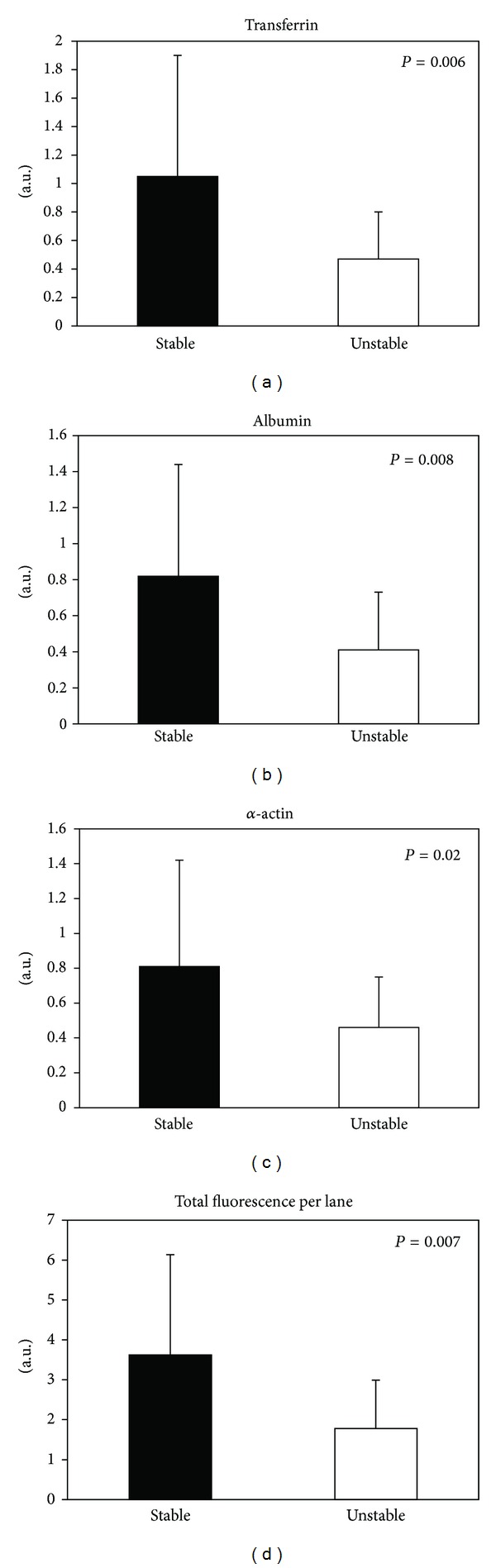
Graphic representation of results obtained by SDS-PAGE of F5M-labelled proteins extracted from stable (black bars) and unstable (empty bars) plaques. The fluorescence intensity signals (arbitrary units) of single bands (a, b, and c) and single lanes (d) were normalized for the corresponding signals obtained after Blue Coomassie G-250 staining.

Differential analysis of F5M-labeled PSH groups by 1D SDS-PAGE indicated deep differences in oxidative state related to plaque stability (Figure 1), whereas no significant difference was observed in plasma samples (data not shown), where only the band corresponding to albumin was detectable. After normalization, the total protein fluorescence signal was significantly different (Figure 2(d)), being approximately twofold lower in unstable plaque extracts than in stable ones (*P* = 0.007). Significant differences were evidenced for transferrin (Figure 2(a), UN/ST = 0.43, *P* = 0.006), albumin (Figure 2(b), UN/ST = 0.48, *P* = 0.008), and *α*-actin (Figure 2(c), UN/ST = 0.56, *P* = 0.02). Since the fluorescent probe used is known to be effective for labeling reduced protein sulfhydryl groups forming a stable thioether bond [16], the reduced fluorescence intensity observed in unstable plaque extracts, compared to stable ones, reflects a more oxidized status of protein-SH groups in the formers. The lack of differences between the corresponding subsets of plasma samples suggests that PSH oxidative modifications mainly take place within the arterial wall.

**Figure 3:**
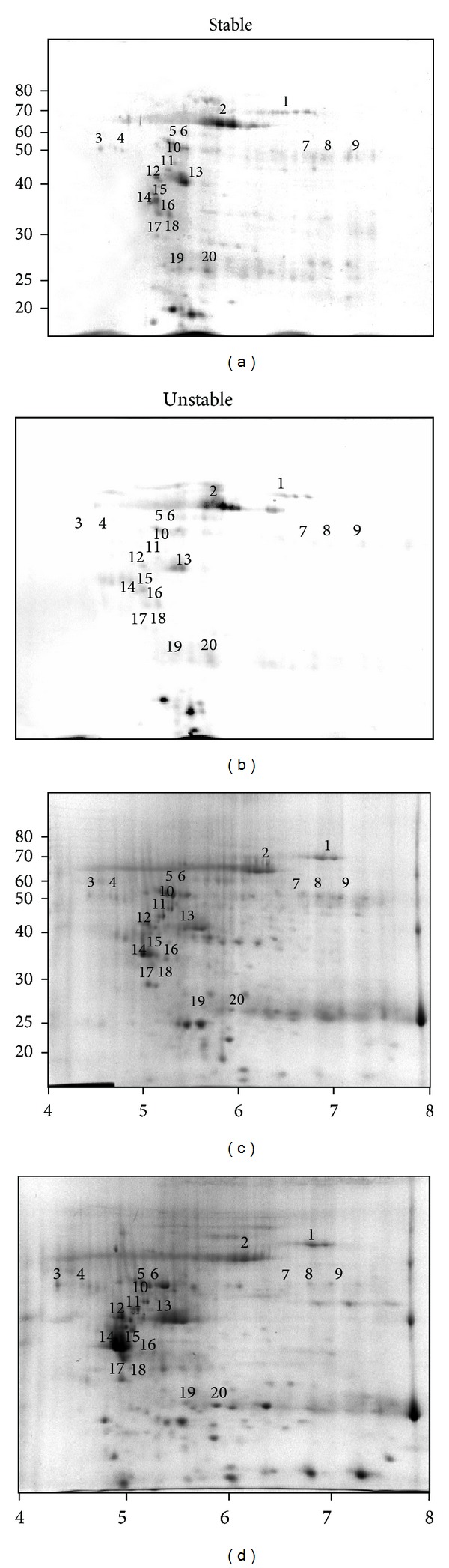
Representative 2D electrophoretic patterns of F5M-labelled proteins and the corresponding CBB staining from stable ((a) and (c)) and unstable ((b) and (d)) plaque extracts. In (a) and (b), inverted fluorescent images are reported. The molecular weight scale was constructed from protein standards (Invitrogen BenchMark Protein Ladder) run alongside the focused strip in the second dimension. The pI scale is based on the linear immobilized pH gradient over 7 cm strips. The numbers indicated on the gels correspond to the spot numbers given in Table 1.

2DE analysis allowed a better identification of involved proteins, also confirming the differences in F5M-labeling between the stable and unstable plaque extracts (Figure 3). A total of fourteen F5M-labeled proteins, either filtered or topically expressed, were identified by MALDI-TOF MS analysis (Table 1). On the basis of our previous proteomics data [4], none of them, with the exception of heat shock protein 27, showed significant differential expression between stable and unstable plaque extracts. Overall, the 2DE results corroborated the finding of a higher protein-SH group oxidation in unstable plaques.

### 3.2. LMW-Thiols Determinations (CZE Analysis)

**Figure 4:**
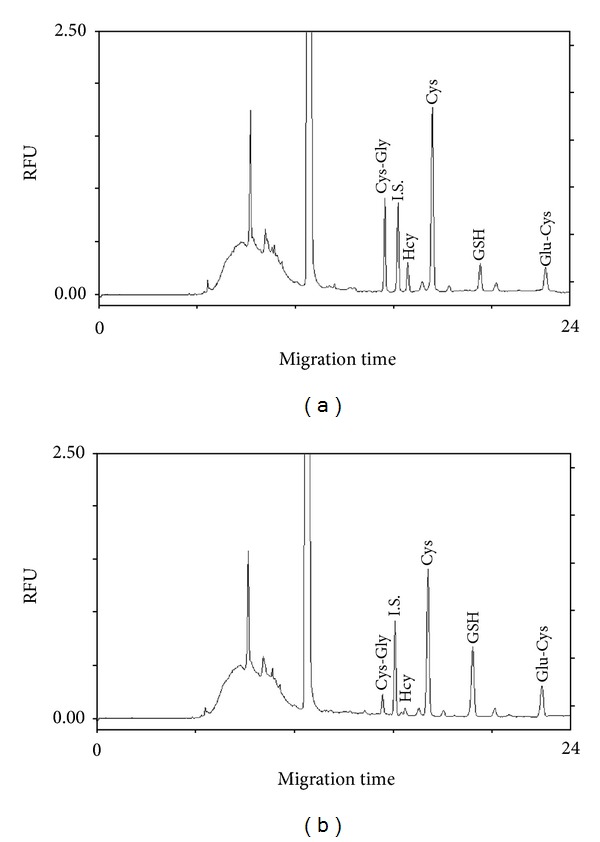
Typical electropherograms of total (a) and protein-bound (b) LMW-thiols from carotid plaque extracts. Cys-Gly: cysteinylglycine; Hcy: homocysteine; Cys: cysteine; GSH: glutathione; Glu-Cys: glutamylcysteine; I.S.: internal standard.

The adopted CZE-LIF method, owing to its elevated sensitivity and selectivity, represents a good tool for an ultrasensitive analysis of LMW-thiols in tissue samples. A representative electropherogram of total and protein-bound LMW-thiols from plaque extracts is shown in Figure 4. Levels of LMW thiols in the atherosclerotic tissue extracts are reported in Table 2. No differences in total LMW thiols were evidenced between stable and unstable plaque extracts. On the contrary, protein-bound LMW thiols levels were significantly higher in unstable than in stable plaque extracts (373 ± 111 versus 283 ± 126 nmol/g prot). The analysis of plasma LMW-thiols did not show differences in both total and protein-bound levels between the two subgroups of patients (data not shown). Moreover, plasma thiols concentration and distribution were similar to those previously obtained in healthy subjects [14]. Interestingly, both levels and distribution of LMW-thiols were significantly different between plasma and arterial tissue extracts confirming our previous findings [15].

## 4. Discussion

A large body of evidences has implicated free radicals and oxidative stress in atherogenesis processes [5, 17]. The endogenous antioxidant capacity of arterial tissues seems to be relevant on this matter since LDL oxidation may occur in sequestered domains of the arterial wall, where a low antioxidant potential and/or a high prooxidant activity could be operative [5, 17–19]. Indeed, it has been shown that human atherosclerotic plaques had low levels of glutathione-related enzyme antioxidant protection [20], so confirming the hypothesis that a specific antioxidant/prooxidant imbalance, operative in the vascular wall, might be involved in atherogenic processes in humans. In this respect, we recently evidenced a reduction in the levels of the enzymes superoxide dismutase 3 and glutathione S-transferase in advanced unstable carotid plaques [4].

Attempting to investigate the relationship between oxidative stress and plaque progression, we studied some posttranslational oxidative modifications of extractable proteins from atherosclerotic plaques by means of a differential proteomic approach. To this extent, we preliminarily investigated protein carbonyl groups and HNE adducts as biomarkers of oxidative stress, without detecting substantial differences between stable and unstable plaques (data not shown).

The present study has provided evidence that in the histologically classified unstable carotid plaques, and to a lesser extent in the stable ones, there is a pro-oxidant microenvironment conducive to the formation of ROS- and RNS-mediated protein thiols oxidation products, as well as of mixed disulfides between proteins and LMW thiols. 

The significant PSH groups oxidation observed regards both filtered and topically expressed proteins. Although several specific proteins showed a different degree of sulfhydryl group oxidation between stable and unstable plaque extracts, the most pronounced differences regarded albumin, *α*-actin, and transferrin. 

Albumin is a nonglycosylated, single-chain polypeptide tightly folded into three domains that are structurally defined by 17 intra-chain disulfide bonds. Albumin Cys^34^, the only cysteine residue uninvolved in intrachain disulfide bonds, accounts for the bulk of free-SH groups in plasma. Its p*K*_*a*_ is abnormally low (~5) compared to that of most plasma LMW-thiols [21]. It is present primarily in the reduced form (mercaptalbumin), although about 30%–40% could be variably oxidized, either reversibly (non-mercaptalbumin) as mixed disulfide with LMW-thiols [22], S-nitroso Cys [23], and sulfenic acid or, irreversibly, as sulfinic or sulfonic acid [24]. Furthermore, it has been described that albumin, through nucleophilic residues, and in particular Cys^34^, is the main plasma target of reactive carbonyl species such as 4-hydroxy-trans-2-nonenal so acting as an endogenous detoxifying agent for these proatherogenic species [25]. Recently, we have developed a new highly sensitive analytical method for the quantification of LMW-thiols bound to both circulating and tissue-retained albumin [26]. Preliminary results suggested that about 35% of filtered albumin carries LMW-thiols showing different thiolation pattern compared with the corresponding circulating form.

Mammalian *α*-actin contains five cysteine residues in the reduced form [27]. Dalle-Donne et al. [27, 28] have shown how a reversible *S*-glutathionylation of Cys^374^ regulated the actin filament formation by inducing structural changes in the actin molecule. 

A human serum purified mature transferrin containing 19 disulfide bonds, and no free sulfhydryl groups have been described [29]. Indeed, we did not evidence any fluorescent signal from plasma transferrin (data not shown), further confirming these observations. However, the evidences we obtained on tissue samples clearly indicate that arterial transferrin is able to bind the fluorescent probe, suggesting the presence of some reduced –SH groups in the protein structure. The stability of cysteine redox state is dictated by the dihedral strain energy of disulfide bond [30]. It is well known that free homocysteine may not only react with free protein sulfhydryl groups but also disrupt critical Cys-Cys disulfide bonds, so damaging the protein structure and compromising its functionality [31]. These reactions are reversible and can vary depending on the redox potentials of the biological systems [32]. We hypothesize that some disulfide bridges may be destroyed following the transferrin infiltration into the prooxidative microenvironment of the subendothelial space leading to the formation of sites for protein mixed-disulfide modifications and probe binding. 

Overall, our proteomic analysis on oxidative modifications of extracted PSH suggested a more pronounced oxidative environment in unstable plaques.

These results were partially confirmed by CZE-LIF analysis of LMW thiols bound to proteins showing higher levels in unstable plaque extracts. Interestingly, such an increase in protein-bound thiols content was not associated with a similar increase in total LMW thiols content. Furthermore, LMW-thiols plasma levels did not discriminate between patients with stable and unstable plaques. 

Results obtained by the CZE analysis, compared with those by proteomics, suggest that in unstable plaques, the higher protein-SH thiolation accounts only partially for the total PSH oxidation observed. In this respect, other PSH oxidation processes, either reversible or irreversible, could be implied. Although intraplaque protein S-thiolation is only one of the possible mechanisms of protein-thiols oxidation, we postulate that the more pronounced S-thiolation in the unstable plaques might have important consequences. It is known that the activity of some MMPs is regulated by thiolation of specific cysteine residues according to a cysteine switch mechanism [11, 12, 33, 34]. The degradation of extracellular matrix by these enzymes is a tightly controlled process under normal circumstances. However, within the atherosclerotic plaque, the balance may be shifted towards matrix degradation, particularly at the rupture-prone shoulder regions of the fibrous cap where accumulating macrophages and phenotypically altered smooth muscle cells secrete a plethora of proteinases, including MMPs [1, 2]. It has been proposed that these enzymes contribute to plaque rupture, and, indeed, we previously evidenced a higher proteolytic environment in unstable plaques regarding the fragmentation of apoB, apo(a), apoE, and members of the proteoglycan (PG) families [3]. Due to the higher degree of protein thiolation observed in unstable plaques, we may postulate that the elevated proteolytic activity found in these tissues could be explained at least partly by the activation of MMPs through an increased S-thiolation of cysteine switch.

## 5. Conclusions

This work describes the extent of oxidative modifications affecting protein-SH groups in atherosclerotic plaques with different vulnerability and the identity of involved proteins. Moreover, the degree of protein mixed-disulfide modifications in relation to atherosclerotic plaque typology is reported. 

The elucidation of the mechanisms of protein thiolation in the plaque environment deserves further studies. The identification of specific protein oxidative modifications and the understanding of their effects on protein function could provide further insight into the relevance of oxidative stress in atherosclerosis.

## Figures and Tables

**Table 1:** Differentially oxidised proteins identified after 2D electrophoresis by peptide mass fingerprinting analysis.

Spot no.	Identified protein	Accession no. (NCBI)	Theo. Mr (KDa)	Theo. pI	Matched peptides	Coverage (%)
1	Transferrin	gi|4557871|	79.31	6.9	17	25
2	Human serum albumin	gi|31615330|	66.44	5.7	14	21
3, 4	Alpha-2HS-glycoprotein	gi|4502005|	40.11	5.4	3	23
5, 6	Alpha-1-antitrypsin	gi|1703025|	46.89	5.4	11	22
7, 8, 9	Fibrin beta	gi|223002|	51.37	8.3	7	21
10	Vimentin	gi|340219|	53.75	5.0	12	32
11	Vimentin	gi|5030431|	41.66	4.8	12	42
12	Vimentin	gi|340234|	35.09	4.7	10	42
13	Alpha actin	gi|4885049|	42.34	5.2	10	36
14	Tropomyosin 1	gi|88927|	33.03	4.6	8	21
15	SP40 (Apo-J)	gi|338305|	37.00	5.7	7	33
16	Modulator of apoptosis 1 (MAP-1)	gi|19923584|	39.72	5.2	3	14
17, 18	Tropomyosin 4	gi|4507651|	28.62	4.7	7	26
19, 20	Heat shock 27 KDa protein 1	gi|4504517|	22.82	6.0	8	44

**Table 2:** Levels of protein-bound and total LMW-thiols from stable and unstable plaque extracts.

	Stable (nmol/g prot)	Unstable (nmol/g prot)	*P* value
Protein-bound LMW-thiols			
Cys-Gly	13.2 (11.3–16.9)	12.1 (8.4–17.1)	0.339
Hcy	5.7 ± 3.2	6.7 ± 4.0	0.468
Cys	146.0 (117.5–211.4)	219.6 (147.6–301.9)	0.089
GSH	41.1 (23.2–77.9)	80.8 (42.0–122.1)	0.168
Glu-Cys	14.1 ± 5.6	15.3 ± 7.0	0.593

Total	283 ± 126	373 ± 111	**0.034**

Total LMW-thiols			
Cys-Gly	53.8 ± 21.1	44.6 ± 19.7	0.205
Hcy	15.0 ± 10.2	20.7 ± 12.3	0.179
Cys	600 ± 276	576 ± 208	0.767
GSH	128.6 (95.1–202.4)	171.2 (97.2–346.4)	0.189
Glu-Cys	69.2 ± 29.4	62.7 ± 29.8	0.534

Total	900 ± 273	960 ± 293	0.555

Values are either mean ± SD or median with ranges (in parenthesis).

LMW-thiols levels were expressed as nmol/g of extracted proteins.

Total LMW-thiols are the sum of free LMW-thiols and protein-bound LMW-thiols.

## References

[1] Lutgens E, van Suylen RJ, Faber BC (2003). Atherosclerotic plaque rupture: local or systemic process?. *Arteriosclerosis, Thrombosis, and Vascular Biology*.

[2] Dollery CM, Libby P (2006). Atherosclerosis and proteinase activation. *Cardiovascular Research*.

[3] Formato M, Farina M, Spirito R (2004). Evidence for a proinflammatory and proteolytic environment in plaques from endarterectomy segments of human carotid arteries. *Arteriosclerosis, Thrombosis, and Vascular Biology*.

[4] Lepedda AJ, Cigliano A, Cherchi GM (2009). A proteomic approach to differentiate histologically classified stable and unstable plaques from human carotid arteries. *Atherosclerosis*.

[5] Steinberg D (1997). Low density lipoprotein oxidation and its pathobiological significance. *Journal of Biological Chemistry*.

[6] Stocker R, Keaney JF (2004). Role of oxidative modifications in atherosclerosis. *Physiological Reviews*.

[7] Eaton P (2006). Protein thiol oxidation in health and disease: techniques for measuring disulfides and related modifications in complex protein mixtures. *Free Radical Biology and Medicine*.

[8] Klatt P, Lamas S (2000). Regulation of protein function by S-glutathiolation in response to oxidative and nitrosative stress. *European Journal of Biochemistry*.

[9] Hogg PJ (2003). Disulfide bonds as switches for protein function. *Trends in Biochemical Sciences*.

[10] Biswas S, Chida AS, Rahman I (2006). Redox modifications of protein-thiols: emerging roles in cell signaling. *Biochemical Pharmacology*.

[11] Bescond A, Augier T, Chareyre C, Garçon D, Hornebeck W, Charpiot P (1999). Influence of homocysteine on matrix metalloproteinase-2: activation and activity. *Biochemical and Biophysical Research Communications*.

[12] Okamoto T, Akaike T, Sawa T, Miyamoto Y, Van der Vliet A, Maeda H (2001). Activation of matrix metalloproteinases by peroxynitrite-induced protein S-glutathiolation via disulfide S-oxide formation. *Journal of Biological Chemistry*.

[13] Zinellu A, Sotgia S, Deiana L, Carru C (2005). Quantification of thiol-containing amino acids linked by disulfides to LDL. *Clinical Chemistry*.

[14] Zinellu A, Zinellu E, Sotgia S (2006). Factors affecting S-homocysteinylation of LDL apoprotein B. *Clinical Chemistry*.

[15] Zinellu A, Lepedda A, Sotgia S (2009). Evaluation of low molecular mass thiols content in carotid atherosclerotic plaques. *Clinical Biochemistry*.

[16] Bigelow DJ, Inesi G (1991). Frequency-domain fluorescence spectroscopy resolves the location of maleimide-directed spectroscopic probes within the tertiary structure of the Ca-ATPase of sarcoplasmic reticulum. *Biochemistry*.

[17] Sugamura K, Keaney JF (2011). Reactive oxygen species in cardiovascular disease. *Free Radical Biology and Medicine*.

[18] Smith C, Mitchinson MJ, Aruoma OI, Halliwell B (1992). Stimulation of lipid peroxidation and hydroxyl-radical generation by the contents of human atherosclerotic lesions. *Biochemical Journal*.

[19] Leake DS, Rankin SM (1990). The oxidative modification of low-density lipoproteins by macrophages. *Biochemical Journal*.

[20] Lapenna D, de Gioia S, Ciofani G (1998). Glutathione-related antioxidant defenses in human atherosclerotic plaques. *Circulation*.

[21] Peters T (1996). *All about Albumin: Biochemistry, Genetics, and Medical Applications*.

[22] Ogasawara Y, Mukai Y, Togawa T, Suzuki T, Tanabe S, Ishii K (2007). Determination of plasma thiol bound to albumin using affinity chromatography and high-performance liquid chromatography with fluorescence detection: ratio of cysteinyl albumin as a possible biomarker of oxidative stress. *Journal of Chromatography B*.

[23] Stamler JS, Jaraki O, Osborne J (1992). Nitric oxide circulates in mammalian plasma primarily as an S-nitroso adduct of serum albumin. *Proceedings of the National Academy of Sciences of the United States of America*.

[24] Carballal S, Alvarez B, Turell L, Botti H, Freeman BA, Radi R (2007). Sulfenic acid in human serum albumin. *Amino Acids*.

[25] Aldini G, Vistoli G, Regazzoni L (2008). Albumin is the main nucleophilic target of human plasma: a protective role against pro-atherogenic electrophilic reactive carbonyl species?. *Chemical Research in Toxicology*.

[26] Zinellu A, Lepedda A, Sotgia S (2010). Albumin-bound low molecular weight thiols analysis in plasma and carotid plaques by CE. *Journal of Separation Science*.

[27] Dalle-Donne I, Giustarini D, Rossi R, Colombo R, Milzani A (2003). Reversible S-glutathionylation of Cys374 regulates actin filament formation by inducing structural changes in the actin molecule. *Free Radical Biology and Medicine*.

[28] Dalle-Donne I, Rossi R, Giustarini D, Colombo R, Milzani A (2003). Actin S-glutathionylation: evidence against a thiol-disulphide exchange mechanism. *Free Radical Biology and Medicine*.

[29] Wada I, Kai M, Imai S, Sakane F, Kanoh H (1997). Promotion of transferrin folding by cyclic interactions with calnexin and calreticulin. *EMBO Journal*.

[30] Schmidt B, Ho L, Hogg PJ (2006). Allosteric disulfide bonds. *Biochemistry*.

[31] Hutchinson S, Aplin RT, Webb H (2005). Molecular effects of homocysteine on cbEGF domain structure: insights into the pathogenesis of homocystinuria. *Journal of Molecular Biology*.

[32] Gilbert HF (1995). Thiol/disulfide exchange equilibria and disulfide bond stability. *Methods in Enzymology*.

[33] Van Wart HE, Birkedal-Hansen H (1990). The cysteine switch: a principle of regulation of metalloproteinase activity with potential applicability to the entire matrix metalloproteinase gene family. *Proceedings of the National Academy of Sciences of the United States of America*.

[34] Chakraborti S, Mandal M, Das S, Mandal A, Chakraborti T (2003). Regulation of matrix metalloproteinases: an overview. *Molecular and Cellular Biochemistry*.

